# Attenuation of stretch-induced arrhythmias following chemical ablation of Purkinje fibres, in isolated rabbit hearts

**DOI:** 10.3389/fphys.2023.1154157

**Published:** 2023-04-06

**Authors:** Miriam Hurley, Richard Walton, Edward J. Vigmond, Michel Haïssaguerre, Olivier Bernus, Ed White

**Affiliations:** ^1^ School of Biomedical Sciences, University of Leeds, Leeds, United Kingdom; ^2^ INSERM Centre de recherche Cardio-Thoracique de Bordeaux, Université Bordeaux, Pessac-Bordeaux, France; ^3^ IHU Liryc, Electrophysiology and Heart Modeling Institute, Fondation, Bordeaux Université, Pessac-Bordeaux, France; ^4^ Electrophysiology and Ablation Unit, Bordeaux University Hospital (CHU), Pessac, France

**Keywords:** ventricular arrhythmias, stretch, endocardium, Purkinje fibres, Lugol, *in silico* modelling

## Abstract

Purkinje fibres (PFs) play an important role in some ventricular arrhythmias and acute ventricular stretch can evoke mechanically-induced arrhythmias. We tested whether Purkinje fibres, play a role in these arrhythmias. Pseudo-ECGs were recorded in isolated, Langendorff-perfused, rabbit hearts in which the left ventricular endocardial surface was also irrigated with Tyrode, *via* an indwelling catheter placed in the left ventricular lumen. The number and period of ectopic activations was measured during left ventricular lumen inflation *via* an indwelling fluid-filled balloon (500 μL added over 2 s and maintained for 15 s in total). Mechanically-induced arrhythmias occurred in 70% of balloon inflations: they were maximal in the first 5 s and ceased within 15 s. Brief, (10 s) irrigation of the left ventricular lumen with Lugol solution (IK/I_2_), *via* the indwelling catheter, reduced inflation-induced ectopics by 98% (*p* < 0.05). Ablation of endocardial PFs by Lugol was confirmed by Triphenyltetrazolium Chloride staining. Optical mapping revealed the left ventricular epicardial activation patterns of ectopics could have PF-mediated and focal sources. *In silico* modelling predicted ectopic sources originating in the endocardial region propagate to and through the Purkinje fibres network. Acute distention-induced ectopics are multi-focal, their attenuation by Lugol, their activation patterns and *in silico* modelling indicate a participation of Purkinje fibres in these arrhythmias.

## 1 Introduction

Mechanical and electrical activity of the heart interact in the processes of excitation-contraction coupling (Bers, 2002) and mechano-electric feedback/coupling (Quinn and Kohl, 2021). The Frank-Starling mechanism describes the positive inotropic effect of stretch on the myocardium (Shiels and White, 2008) while mechanically-activated arrhythmias are an example of mechanical modulation of cardiac electrical activity (Quinn and Kohl, 2021).

The myocardium is an established source of mechanically-activated arrhythmias, for example, in a model of *commotio cordis*, compression of the epicardial myocardium caused ectopic activity, arising from the point of compression (Quinn et al., 2017) and studies have shown that mechanical stimulation of single cardiac myocytes can trigger action potentials (Kohl et al., 2011).

Purkinje fibres (PFs) are recognised as important components of some arrhythmia induction and maintenance (Haïssaguerre et al., 2002; Haïssaguerre et al., 2016) with PF ablation a clinical intervention (Samo Ayou et al., 2014). However, in the field of mechano-electric coupling, PFs are less studied than the myocardium, although studies have shown PFs are sensitive to stretch (Kaufmann and Theophile, 1967; Dominguez and Fozzard, 1979). Therefore, the thin, cable-like properties of PFs, with low sink-source barriers to conduction (compared with myocardium) and branching network of conductive tissue (Campos et al., 2015; Campos et al., 2017) make them credible contributors to some types of mechanically-activated arrhythmias. The aim of our study was to investigate this possibility.

A commonly used experimental technique to investigate responses to mechanical stimulation is acute cavity dilation/distension in isolated hearts, by inflation of an indwelling fluid-filled balloon. This dilates the chamber and can provoke ectopic activity (Dick and Lab, 1998; Bode et al., 2001). Chamber dilation stretches PFs lining the endocardial surface of the ventricles (Canale et al., 1983), thus this model was appropriate to study a potential role for PFs in these stretch-induced arrhythmias. This technique may create a mechanical stimuli that, in addition to increased axial strain, may include compression and shear, the term ‘stretch’ is used to encompass all mechanical stimuli. We chose the rabbit, as this species is often used in studies of mechano-electric coupling (Quinn and Kohl, 2016). We used brief exposure to Lugol solution (KI/I_2_) as a well-established technique to study the effect of chemical ablation of PFs on cardiac electrical activity (Damiano et al., 1986; Chen et al., 1993; Cha et al., 1995; Dosdall et al., 2008; Myles et al., 2012). Optical mapping was used to investigate the electrical activation profiles of acute dilation-induced arrhythmias because activation profile carries information regarding the source of stimulation; activation by PF stimulation has a broader and faster spread than that mediated by focal myocardial stimulation (Martinez et al., 2018). *In silico* modelling with a finite element model (Behradfar et al., 2014) was used to predict the propagation of ectopics arising from the endocardial region.

## 2 Methods

All experiments were performed according to the United Kingdom Animals (Scientific Procedures) Act of 1986, and local ethical approval (AWERC211701EW). Male New Zealand White Rabbits (2.5–3.0 kg, N = 12) were euthanised by a subcutaneous injection of 10 mg/kg Ketamine (Ketavet, Zoetis) and 3 mg/kg Xylazine (Rompun 2% solution, Bayer), followed by an intravenous injection of 200 mg/mL Pentobarbitone (Pentoject, Animalcare). Hearts were quickly excised, cannulated at the aorta and perfused with ice-cold cardioplegic solution, containing (in mM); Glucose 277.5, KCl 30, NaHCO_3_ 25, Mannitol 34.3 with 5 U/mL Heparin, (pH 7.4) until the coronary circulation had cleared. Hearts were weighed then transferred to a Langendorff perfusion system where Tyrode solution containing (in mM); NaCl 130, NaHCO_3_ 24, NaH_2_PO_4_ 1.2, MgCl_2_ 1, Glucose 5.6, KCl 4, CaCl_2_ 1.8, oxygenated with 95% O_2_ and 5% CO_2_ to maintain a pH 7.4, was perfused at 38°C at a rate of 20 mL/min. All chemicals were purchased from Sigma.

### 2.1 LV balloon placement and LV lumen irrigation in contracting hearts

A cellophane balloon was placed in the left ventricle (LV) lumen. Access to the LV was gained through partial dissection of the left atrium, allowing for entry *via* the mitral valve. This route served as drainage for the LV. The balloon formed part of a fluid filled closed system that included metal tubing (inner diameter 1 mm) connecting to a pressure transducer and syringe. The balloon was stabilised in the LV lumen by securing the tubing to the aortic cannula. Injecting or withdrawing fluid from the syringe allowed the balloon to be inflated or deflated. An additional cannula (inner diameter 1 mm) was placed in the LV lumen, *via* the same route as the balloon, to provide constant irrigation of the LV luminal surface with Tyrode or brief irrigation with Lugol (see section 2.2). Thus, the whole heart was Langendorff perfused *via* the aorta with Tyrode and the LV lumen was irrigated with Tyrode or briefly with Lugol. There was no recirculation of solutions.

### 2.2 Experimental design and data analysis in contracting hearts

Mechanical stimulation was undertaken whilst the hearts were in sinus rhythm to maintain the physiological excitation pathway of His/PFs to myocardium. No electrical stimulation protocols were used to elicit ectopics. Needle electrodes were used to record a pseudo lead II ECG, with the negative electrode placed at the right atrium and positive electrode placed at the ventricular apex.

To stretch the LV, the fully deflated balloon was inflated until a stable diastolic pressure in the range of 2–6 mm Hg was reached and a left ventricular developed pressure (LVDP, an index of contractility) of approximately 10 mmHg was recorded. This was achieved by injecting a volume of 0.2 mL. LV stretch was elicited by injecting an additional 0.7 mL into the balloon over 2 s to reach a final inflated volume of 0.9 mL. Inflation was maintained for 13 s prior to the removal of 0.7 mL fluid. Preliminary studies showed these volumes were close to the bottom and top of the ascending Frank-Starling relationship for hearts of this size.

The LV was stretched 3 times, with 90 s between each stretch (Dick and Lab, 1998). The Tyrode irrigation of the endocardial surface of the LV was then changed to irrigation with a 0.5 mL volume of Lugol solution (120 mM KI, 38.6 mM I_2_), delivered over a 10 s period, followed by return to continuous irrigation of the LV lumen with Tyrode. Flushing of Lugol from the LV lumen was indicated by a change in LV effluent colour from deep orange (Lugol) to colourless (Tyrode). LV stretch was then repeated 3 times with 90 s between each stretch.

Data were acquired at 1 kHz and analysed with LabChart (ADInstruments). Data was analysed 15 s prior to the initiation of stretch and for 15 s upon the initiation of stretch, this period captured all ectopic activity. From the pseudo-ECG the number of non-sinus beats and their duration were calculated. Ectopic activations were identified by their waveform and timing, relative to sinus activations. The duration of ectopics was the time from the first ectopic beat to the first sinus beat after the last ectopic. The pseudo-ECG was also used to measure heart rate. Pressures were measured at steady state at both balloon volumes. Changes in heart rate and LVDP were used to assess changes in basal electrical and mechanical activity caused by Lugol.

### 2.3 Optical mapping in non-contracting hearts

Optical mapping was performed in 4 hearts. The hearts were Langendorff perfused *via* the aorta with a 1 L volume of recirculating Tyrode at 20 ml/min at 38°C. To prevent motion artefacts, a single 25 mL, 86 μM bolus of Blebbistatin (BioServ), dissolved in DMSO and diluted in Tyrode was perfused, followed by a 25 mL, 72 μM bolus of the potentiometric dye RH237 (ThermoFisher), the effluent of which was collected and perfused a second time. To avoid over perfusion of the heart, the Langendorff perfusion pump was stopped whilst Blebbistatin and RH237 were added *via* a port at the entrance to the aortic cannula’s heating coil.

Following addition of dyes the hearts were lowered into an experimental chamber and orientated to ensure that the LV epicardial surface was facing the mapping system. RH237 was excited by 530 nm light from 4 LEDs. RH237 fluorescence was detected using a CCD camera (Scimeasure) and relevant filters at 685 nm (Cairn Research).

Epicardial activation was recorded during sinus stimulation, to record activation in response to stimulation conducted through the Purkinje system. This was contrasted with activation in response to focal endomyocardial stimulation which was elicited *via* external platinum electrodes placed at the base of the LV endocardium. Recordings were also obtained in response to mechanical stimulation of the LV upon inflation of a LV indwelling fluid-filled balloon, as previously described. No electrical stimulation protocols were used to elicit ectopics. A pseudo-ECG record was made simultaneously *via* non-contact electrodes within the experimental chamber.

To test the effect of stretch and Lugol on action potential (AP) parameters, following the above procedures, the hearts were lifted from the experimental chamber and a cannula placed in the LV lumen, through which 0.5 mL of Lugol was injected over a 10 s period, this was followed by irrigation with Tyrode until the LV effluent ran clear. Lugol effluent was collected and not allowed to enter the recirculating Tyrode. The heart was then lowered back into the experimental chamber to allow optical measurements to be made as described above.

Optical mapping data was acquired at 1 kHz with an 80*80 pixel, 26 mm square field of view using Turbo software (Scimeasure). Epicardial activation maps were created with BV analysis (Brainvision) based on the time from the start of the earliest AP upstroke to 50% peak AP height in a given pixel, using 3*3 spatial and cubic filters. Maps were colour-coded with 4 ms contours. BV analysis was also used to measure mean AP duration (APD) at 20% and 80% repolarisation from individual pixels with the SD of APD80 used as a measure of repolarisation dispersion. Pseudo-ECG data were acquired at 1 kHz and analysed with LabChart.

### 2.4 Assessment of Lugol tissue damage

Triphenyltetrazolium Chloride (TTC; Merck, T8877) was used to identify the extent of tissue necrosis caused by brief exposure to Lugol (Myles et al., 2012). Following functional experiments involving Lugol exposure, the LV and RV free wall were cut to reveal the LV and RV luminal cavities. A TTC solution (29.87 mM TTC, 135.15 mM Trizma-HCl, 69.34 mM Trizma base) was warmed to 37°C and the heart immersed for 40 min at 20°C–22°C. Afterwards, the heart was fixed with 10% formalin solution (4% paraformaldehyde, Sigma) for 60 min at 20°C–22°C. Images of the endocardial surface of the LV and RV were acquired using a Sony cyber-shot digital camera, with healthy muscle exhibiting a dark red appearance which contrasted with the white appearance of necrotic tissue.

### 2.5 *In silico* experiments

A previously detailed finite element model of rabbit ventricles incorporating a realistic branching PF network and myocardial fibre anisotropy (Behradfar et al., 2014; Campos et al., 2015; Campos et al., 2017) was used to predict the propagation of PF and myocardial ectopic sources from 32 putative ectopic sites within the LV, these were chosen by the researchers to give a wide coverage of the LV. The position of these sites is given in Supplementary Figure S1 and the resultant epicardial activation patterns of the 64 simulations in Supplementary Figure S2 (see supplementary Methods and Results for further details).

### 2.6 Data presentation and statistical analysis

Data are given as population mean ± SEM from 8 hearts prior to Lugol exposure (Tyrode) and 7 hearts after Lugol exposure (Lugol) in contracting hearts or from 4 hearts in the optical mapping study. Statistical analysis was performed with Sigmastat 3.5. Following data normality testing, either the paired Student's t-test or the non-parametric equivalent, Wilcoxon signed rank test, was performed on paired data from 7 contracting hearts, using the mean of each parameter in each condition (Tyrode vs. Lugol). Statistical significance was taken as *p* < 0.05.

Alternative analysis where n was individual paired stretches (with the 1^st^ 2^nd^ and 3^rd^ stretch in each Tyrode sequence paired with the equivalent Lugol stretch in each heart) resulted in the same statistical outcomes with respect to *p* < or >0.05.

For pressure and AP parameters the effects and possible interactions of stretch and Lugol exposure were tested by 2 way analysis of variance (2 way ANOVA) with balloon volume (stretch) and exposure to Lugol as the 2 factors. The parameter means from each heart in each condition were used. A result of *p* < 0.05 for either factor or factor interaction was followed by Holm-Sidak multiple pairwise comparision tests.

## 3 Results

**FIGURE 1:**
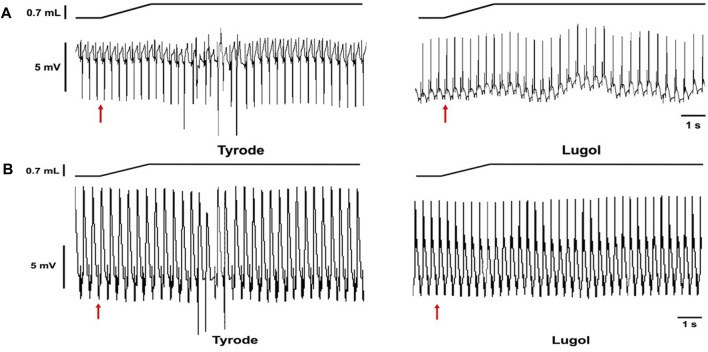
Stretch-induced ectopics are attenuated by Lugol. Records from 2 Langendorff perfused rabbit hearts in sinus rhythm **(A,B)**. Schematic of change in balloon volume (upper traces) and pseudo-ECG (lower traces). The red arrows indicates the start of a left ventricular (LV) indwelling balloon inflation from 0.2 mL to 0.9 mL to dilate/stretch the LV. Records prior to Lugol treatment (left, Tyrode) and following LV lumen irrigation with 0.5 mL Lugol, over a 10 s period, followed by Tyrode irrigation (right, Lugol). In both hearts, prior to Lugol, dilation provoked mechanically-activated ectopics; following Lugol these ectopics were abolished.

**FIGURE 2:**
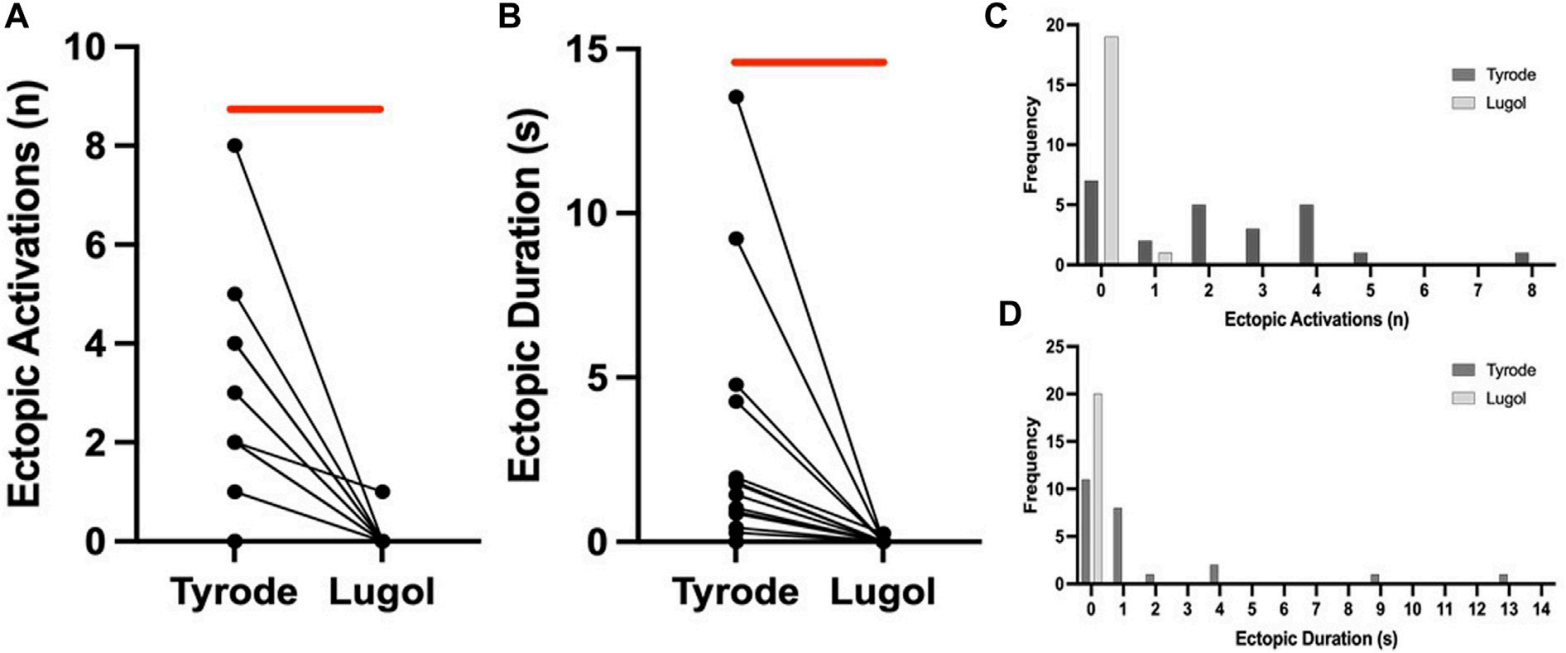
Lugol decreases the number and duration of stretch-induced ectopics. Number of ectopics **(A)** and duration of ectopics **(B)** for paired stretches before (Tyrode) and after (Lugol) exposure to Lugol. After exposure to Lugol, a significant reduction in the number and duration of mechanically-activated ectopics was recorded (red bar). Data from 20 paired stretches from 7 hearts, Wilcoxon signed ranks test for ectopic number and paired Student’s t-test for duration. With either n = 20 stretches or n = 7 hearts (Tyrode vs. Lugol, *p* < 0.05), symbols may represent more than 1 pair of data. Frequency histograms for ectopic number **(C)** and duration **(D)**, a leftwards shift in both parameters is visible after exposure to Lugol, 24 stretches from 8 hearts in Tyrode and 20 stretches from 7 hearts for Lugol.

In Langendorff perfused hearts with Tyrode irrigation of the LV lumen (Tyrode), when a balloon was inflated in the LV lumen from a volume of 0.2 mL–0.9 mL, multifocal ectopic activations were elicited in 17/24 stretches. Ectopics began shortly after dilation commenced and spontaneously ceased within 15s, examples are shown from 2 hearts in Figure 1. Following a 10s irrigation of the LV lumen with Lugol, followed by return to Tyrode irrigation (Lugol), dilation-induced ectopic activations were attenuated (Figure 1 and Figure 2). Lugol reduced the number of ectopic activations by 98% (mean ectopics/stretch: 2.25 ± 0.42, Tyrode vs. 0.05 ± 0.05, Lugol) and ectopic duration by 99% (mean duration: 2.22 s ± 0.78, Tyrode vs. 0.01 s ± 0.01 Lugol), *p* < 0.05, Tyrode data from 8 hearts, Lugol data from 7 hearts.

**FIGURE 3:**
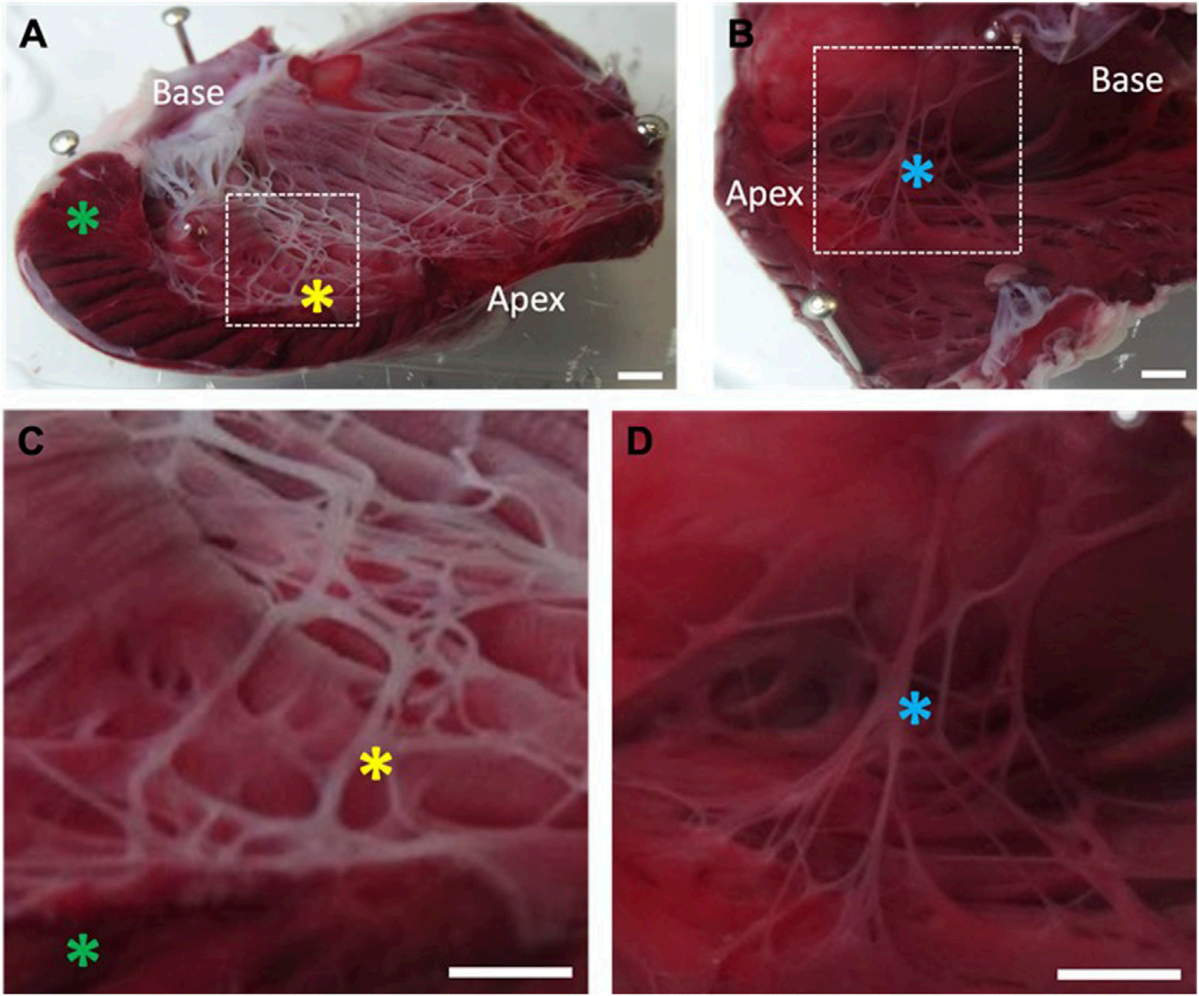
Location of necrotic tissue on the left ventricular endocardial surface following exposure to Lugol. The left ventricular (LV) lumen of isolated Langendorff perfused rabbit hearts were exposed to 0.5 mL Lugol (120 mM KI, 38.6 mM I_2_) solution for 10 s, followed by a Tyrode wash. Hearts were stained with TTC and fixed in 10% formalin prior to imaging. **(A)** LV, with incision through the free wall. The Purkinje fibre network stains as a white meshwork indicating necrosis. The endocardial surface shows a thin layer of necrosis that is not universal, while the LV bulk myocardium is unaffected (green asterick). Prominent, ablated Purkinje fibres are visible (yellow asterick). **(B)** Absence of necrotic tissue on the untreated RV endocardial surface from the same heart, with Purkinje fibres staining as healthy tissue (blue asterick). **(C,D)** Magnified view of the area indicated by dashed box in **(A,B)** respectively. Individual Purkinje fibres are necrotic after exposure to Lugol (C; yellow asterick), but intact within the RV (D; blue asterick) in the absence of Lugol. Scale bars, **(A–D)**: 2 mm.

Following functional experiments LV and RV lumens were stained with TTC to investigate the site of Lugol damage. Figures 3A, C shows the LV PF network stained white, indicating widespread necrosis. There was superficial necrosis on the endocardial surface but some areas remained red, indicating healthy tissue. At the cut edge of the ventricle there was minimal penetration of necrosis into the underlying myocardium. In the RV of the same heart there was no evidence of necrosis in either the PF network or the myocardium (Figure 3B). Thus, in agreement with its use in other studies, the primary target of Lugol was the PF network.

In support of the histological observations, Table 1 shows Lugol had no effect upon the diastolic pressure or LVDP at either balloon volume, indicating no effect upon the contractility of the heart and thus upon the bulk myocardium. Furthermore, following Lugol application, we were able to pace hearts by electrical stimulation of the basal endomyocardium, indicating viable myocardium in this region. However, Lugol significantly (*p* < 0.05) increased the QRS interval of the pseudo-ECG, consistent with a slowing of electrical activation caused by ablation of PFs. A 6% (*p* < 0.05) decrease in heart rate following exposure to Lugol was observed, this may reflect a time-dependent slowing of the preparations as Lugol did not come into contact with the sinus node or cause dropped ventricular beats.

**TABLE 1:** Langendorff perfused isolated rabbit hearts in sinus rhythm had an indwelling balloon placed within the left ventricle (LV) that was inflated to an initial volume of 0.2 mL then inflated to a volume of 0.9 mL to dilate/stretch the LV. The LV was irrigated with Tyrode followed by 0.5 mL Lugol (120 mM KI, 38.6 mM I_2_) over 10 s prior to a Tyrode wash. Lugol decreased heart rate and prolonged the QRS interval of the pseudo-ECG. Prolonged QRS is an expected consequence of Purkinje fibre ablation. Diastolic pressure and left ventricular developed pressure (LVDP, an index of contractility) were not altered by exposure to Lugol, indicating a lack of effect upon the contractile response to stretch. Data are mean ± SEM from 8 hearts and 24 stretches for Tyrode and 7 hearts and 21 stretches for Lugol, Statistical analysis of heart rate and QRS was by paired *t*-test on the means from each heart. The effects of stretch and Lugol on pressures was tested by 2 way ANOVA, with stretch and Lugol as factors. There was a significant effect of stretch (*p* < 0.05) but not Lugol or factor interaction (*p* > 0.05). There was a significant effect of stretch in all Holm-Sidak multiple pairwise comparisons.

	Tyrode	Lugol
Hearts(n)	8	7
Heart rate (bpm)	166.70 ± 9.17	155.68 ± 10.87*
QRS (ms)	34.9 ± 0.54	48.5 ± 1.07
Diastolic pressure @ 0.2 mL (mmHg)	2.98 ± 1.05	3.02 ± 1.07
LV developed pressure @ 0.2 mL (mmHg)	10.77 ± 1.71	11.03 ± 1.27
Diastolic pressure @ 0.9 mL (mmHg)	9.56 ± 0.72	9.86 ± 0.85
LV developed pressure @ 0.9 mL (mmHg)	22.48 ± 1.48	25.72 ± 1.86

Optical mapping was used to further investigate the nature of dilation-induced ectopics. Table 2 shows there was no effect of either stretch or Lugol upon the APD20, APD80 or repolarisation dispersion. Therefore stretch (and Lugol) did not cause APD triangulation or increase the, potentially arrhythmogenic, dispersion of repolarisation (see Discussion).

**TABLE 2:** Langendorff perfused isolated rabbit hearts in sinus rhythm had an indwelling balloon placed within the left ventricle (LV) inflated initially to a volume of 0.2 mL (Relaxed) then inflated to 0.9 mL (Stretch) to dilate/stretch the LV. The LV was irrigated with 0.5 mL Lugol (120 mM KI, 38.6 mM I_2_) over 10 s followed by a Tyrode wash. All data were recorded in sinus rhythm. Neither Lugol or stretch had an effect upon action potential duration at 20% repolarisation (APD20) or 80% repolarisation (APD80), thus, there was no evidence of action potential triangulation. Neither stretch or Lugol affected repolarisation dispersion. Data are mean ± SEM from 4 hearts. Statistical significance was tested with 2 way ANOVA with stretch and Lugol exposure as the 2 factors, *p* > 0.05 for both factors and factor interaction for all parameters.

	Tyrode		Lugol	
	Relaxed	Stretch	Relaxed	Stretch
Hearts (n)	4	4	4	4
APD20 (ms)	36.28 ± 2.47	32.36 ± 1.93	31.27 ± 1.02	29.52 ± 0.89
APD80 (ms)	138.04 ± 3.24	134.73 ± 3.60	136.58 ± 1.47	136.58 ± 1.29
Repolarisation dispersion (ms)	50.38 ± 2.35	47.72 ± 1.37	46.72 ± 1.44	45.67 ± 1.39

**FIGURE 4:**
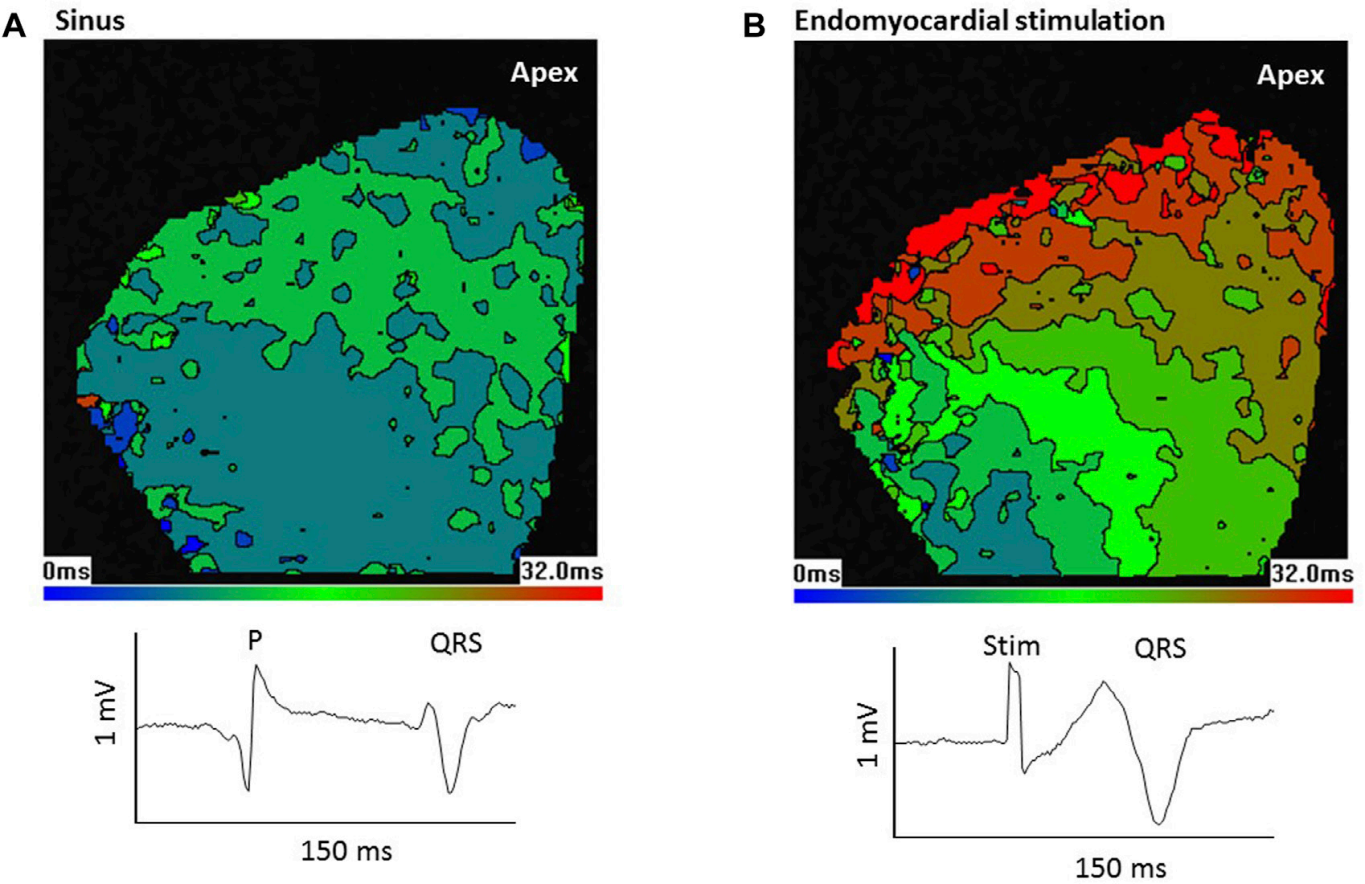
Epicardial activation from sinus stimulation spreads faster than from endomyocardial stimulation. Optical mapping of left ventricular (LV) epicardial activation in response to **(A)** sinus stimulation or **(B)** focal endomyocardial stimulation near the LV base. Images show colour coded 4 ms contours. Images are 80 × 80 pixels in 26 mm squares. In response to sinus stimulation, which involves conduction through the Purkinje network, early activation occurs at the base and apex and there is a rapid spread of activation. In contrast focal endomyocardial stimulation results in a small basal area of initial activation that spreads slowly to the apex. Thus, the activation pattern gives information about the stimulation source. Lower traces show sections from the pseudo-ECG; P wave and QRS in sinus and stimulus artefact (Stim) and QRS in endomyocardial stimulation. Consistent with the slower epicardial activation, QRS is longer following endomyocardial stimulation.

Optical mapping of epicardial activation was used to assess the source of endocardial stimulation (Martinez et al., 2018). Figure 4 shows the LV epicardial activation map in response to sinus stimulation (Figure 4A) and, in the same heart, in response to electrical stimulation of the LV basal endomyocardial surface (Figure 4B). The maps are colour-coded in 4 ms contours representing the time taken for activation (AP upstroke) to occur (see Methods). In this heart, in sinus (Figure 4A), early activation was broad, occurred at both the base and apex and spread quickly over the epicardial surface. In contrast, with endomyocardial stimulation (Figure 4B), early activation was restricted to a small area in the base which then spread slowly towards the apex. Below the activation maps, the QRS waveforms from the pseudo-ECG show lengthening with endomyocardial stimulation, this was seen in all 4 hearts tested.

**FIGURE 5:**
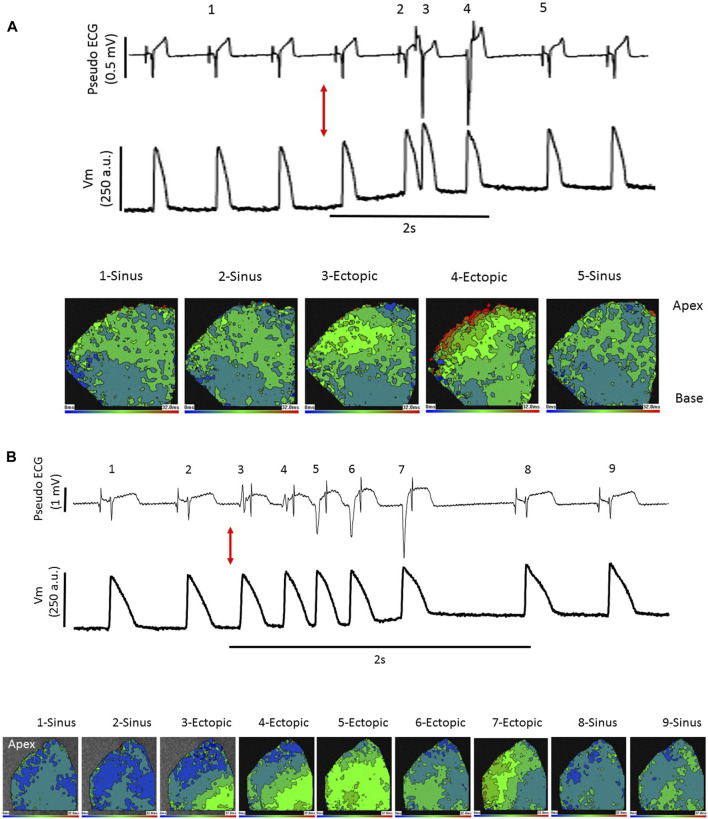
Stretch-induced ectopic activation mimics sinus and focal stimulation. Pseudo-ECG (upper trace), optically measured action potentials (middle trace) and epicardial activation maps, as described in Figure 4 (lower images), in the left ventricle (LV) of 2 Langendorff perfused rabbit hearts **(A, B)**. **(A)** at the red arrow the LV is dilated from 0.2 mL to 0.9 mL by inflation of an indwelling balloon over a 2 s period, triggering ectopics. Activation maps from selected points 1–5 show sinus stimulations at 1, 2 and 5 with broad basal and apical early activation and typical rapid activation spread. Excitations 3 and 4 are ectopic. Activation 3 falls upon the t-wave of the preceding sinus beat but this does not trigger sustained arrhythmias, the activation pattern closely resembles that of sinus stimulation. There is some slowing of lateral conduction, possibly explained by the short preceding diastolic period. Excitation 4 has 2 focal-like early activation sites, in the base and laterally, and conduction is slower than the other 4 excitations. **(B)** at the red arrow the LV is dilated from 0.2 mL to 0.9 mL by inflation of an indwelling balloon over a 2 s period, triggering ectopics. A sequence of 2 sinus excitations 1 and 2 followed by 5 self-terminating ectopic excitations 3–7 and the following 2 re-established sinus excitations 8 and 9 are shown. In this heart sinus excitation leads to lateral early activation. This is mimicked by ectopics 3 and 6 but ectopics 4, 5 and 7 have distinct patterns. Dilation-induced ectopics are multi-focal and typically within a single ectopic period, there are varied activation patterns.

Because sinus stimulation involves conduction through the PF network, it was predicted that if stretch-induced ectopics were conducted through the PF network, subsequent epicardial activation would resemble aspects of sinus activation patterns. Figure 5A shows pseudo-ECG traces and optically mapped action potentials during a LV stretch. The offset in the optical baseline caused by stretch is 3 orders of magnitude slower than the AP upstroke and has little effect on the calculation of epicardial activation time. Activation maps from 5 selected excitations are presented, in sinus rhythm [1, 2 and 5] epicardial activation originated both basally and apically. Stretch provoked 2 ectopic beats, the first [3] falling on the t-wave of the preceding activation (see Discussion). Although some lateral conduction was slowed, this activation manifested distinct similarities to sinus stimulation [1, 2 and 5] such as the basal and apical activation origins, strongly indicating a PF involvement. However, the following ectopic [4] differs, demonstrating focal basal and lateral activation origins with more pronounced conduction slowing. Figure 5B shows a similar experiment from another heart, here sinus activation began laterally [1, 2, 8, 9] and a run of 5 ectopics [3–7] showed varied activation patterns, with [3] demonstrating rapid activation spread and [6] the bi-lateral activation pattern seen in sinus, but others [4, 5 and 7] displaying individual activation patterns. In the 4 hearts tested a consistent observation was that initial ectopics showed the same regional origins as sinus activations, these origins could then change during an ectopic run. Ectopic breakthroughs with regional similarity to sinus activation but slowed conduction might be explained by a shorter preceding diastolic interval or partial activation of the PF network.

Despite the change in the mechanical state of the myocardium following blebbistatin treatment, the frequency of ectopics was similar to that recorded in contracting hearts. In contracting hearts stretch caused eptopics in 71% of stretches with a mean frequency of 2.25 ± 0.42 ectopics per stretch (24 stretches from 8 hearts). In hearts treated with blebbistatin, stretch caused ectopics in 70% of stretches with a mean frequency of 2.9 ± 0.85 ectopics per stretch (10 stretches from 4 hearts).

**FIGURE 6:**
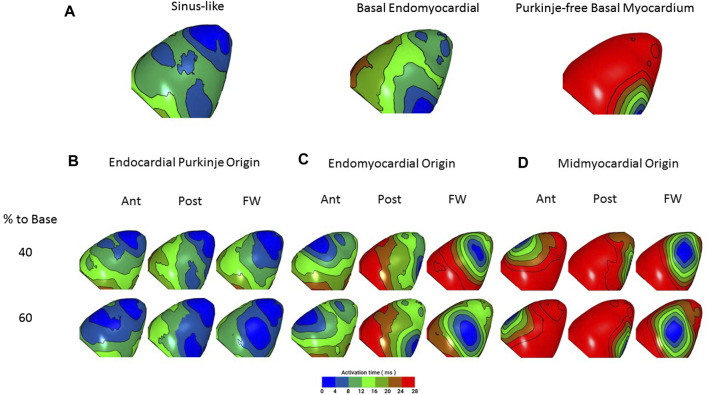
*In silico* simulation of epicardial activation in response to ectopic foci. Hearts are orientated with apex to the top to match experimental images. Epicardial activation is colour-coded as in the optical studies (Figures 4, 5). **(A)** Left, closest match to sinus stimulation (deep foci, PF origin 40% to base). Centre, match to basal endomyocardial stimulation (superficial foci, myocardial origin, 80% to base). Right, basal myocardial stimulation from a ‘PF-free’ region (deep foci, myocardial origin, 80% to base). There is close similarity between the characteristics of the activation patterns predicted by sinus and endomyocardial stimulation and those optically recorded (see Figure 5). Sinus stimulation leads to broader initial breakthrough and a faster spread of activation. Note that endomyocardial stimulation that does not include PF propagation results in activation slower than that seen in Figure 4B. **(B–D)** Epicardial activation in response to ectopic foci (arising, for example from mechanical stimulation) in the LV anterior (Ant), posterior (Post) and free wall (Free) at 40% and 60% distance to the LV base. **(B)** foci with an endocardial PF origin, **(C)** Foci with an endomyocardial origin, **(D)** Foci with a midmyocardial origin. Activation spread is fastest from PF origins then from the endomyocardium and slowest from the midmyocardium. When compared with ectopic activations presented in Figures 5A,B, closest matches are with PF and endomyocardial origins (consistent with Lugol ablation of these ectopics). Crucially, the model predicts that ectopic depolarisation arising in the endomyocardium will migrate to and propagate through PFs, resulting in a faster spread of activation across the epicardial surface than is seen with midmyocardial (PF-poor) foci. For a full 64 site simulation see Supplementary Figure S2.

*In silico* experiments revealed that epicardial activation resulting from Purkinje origins with deep foci (thus, directly stimulating the His bundle branches) had patterns similar to those seen in optical recordings following sinus stimulation. Endomyocardial foci resulted in slower epicardial spread, in accord with optical recordings (compare Figures 4, 6A).

**FIGURE 7:**
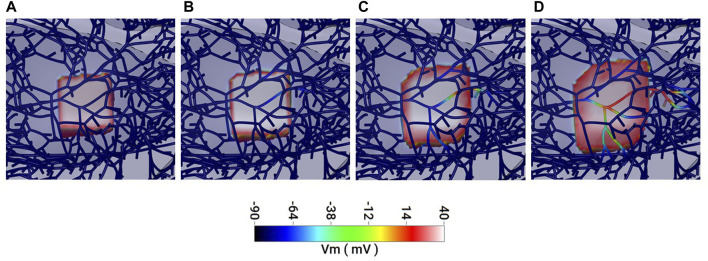
*In silico* simulation of the spread of activation in the myocardium and Purkinje fibre network. **(A–D)** 1 ms sequential frames representing an area of myocardium and associated Purkinje network (dark blue strands in A). **(A)** Myocardium, only, within a 2 mm cubic region is activated (pink/red) and the depolarisation spreads outwards through **(B)** to **(D)**. **(B, C)** Some Purkinje fibres within the depolarised region become activated retrogradely and conduction through these fibres propagates. **(D)** Faster conduction in the Purkinje fibres results in Purkinje-mediated propagation outpacing that from the myocardium. See Supplementary Figure S3 for a video of this effect in the full model.

Figures 6B–D compares epicardial activation when ectopic foci occur in either endocardial PFs (Figure 6B), endocardial myocardium (Figure 6C) or deep endomyocardium in areas lacking PFs (Figure 6D). The epicardial activation patterns most similar to those recorded optically are those with ectopic foci from the endocardial PFs and endomyocardium rather than the deep myocardium (compare Figures 6B–D with Figure 5). Crucially, the model predicts that foci in the endomyocardium will activate proximal PF-muscle junctions and that conduction, propagating through the PF network, will outpace that travelling through the myocardium (Figure 7). Supplementary Figure S3 provides a video of this effect in the whole, dual, ventricle model.

## 4 Discussion

Our novel findings are that, in rabbit hearts, stretch-induced ectopics can display epicardial activation characteristics that mimic sinus (PF-mediated) activations and these ectopics are attenuated by brief exposure of the LV endocardial surface to Lugol, which preferentially targets PFs. These findings indicate that PFs play a role in these stretch-induced ectopics. In accord with these observations, *in silico* modelling predicts that, whether the ectopic focus is in the PF or endomyocardium, propagation will involve the PF network.

The isolated rabbit heart is an established model for the study of mechano-electric interactions (Quinn and Kohl, 2016). Acute ventricular distension has been shown to decrease the effective refractory period, in both Langendorff perfused hearts (Reiter et al., 1988) and isolated working hearts (Halperin et al., 1993) and to decrease the fibrillation threshold (Jalal et al., 1992). Other studies in rabbit have triggered single excitations by single, brief stretches (Franz et al., 1992) or provoked self-terminating runs of ectopics, similar to those we describe (Dick and Lab, 1998). Differences between the findings of these studies are likely linked to experimental conditions as the amplitude, speed and duration of stretch are known to influence the electrical outcomes (White et al., 2005). Ventricular dilation can increase PF strain (Canale et al., 1983), possibly by a combination of fibre unbuckling and sarcolemmal unfolding (Dominguez and Fozzard, 1979).

### 4.1 Lugol preferentially targets PF

Lugol is used to preferentially ablate PFs with minimal effect upon the underlying myocardium (due to the high surface area:volume in cable-like PFs) and so modify electrical activation e.g., (Damiano et al., 1986; Chen et al., 1993; Cha et al., 1995; Dosdall et al., 2008; Myles et al., 2012). We provide TTC staining evidence for PF necrosis and recorded a widening of the QRS, characteristic of the effect of PF ablation on ventricular activation (Damiano et al., 1986; Dosdall et al., 2008; Myles et al., 2012). In contrast, following Lugol, TTC staining revealed areas of healthy endocardial surface and it was possible to pace the heart from the basal endomyocardium, which also indicates viable myocardium. There was no change in LVDP, at deflated or inflated balloon volumes, indicating no effect on bulk myocardial contractility or the Frank-Starling mechanism (Shiels and White, 2008).

Other tissue types at the endocardial surface will be susceptible to Lugol, e.g., autonomic nerve terminals (Drake-Holland et al., 2001), though the signalling pathways activated by stretch in these tissues is slower than the electrical response to stretch we describe. We conclude that, in accord with previous studies, the principal target of Lugol was the PF network, rather than the myocardium.

### 4.2 The profile of ectopic activations

Some studies report stretch-induced triangulation of the myocardial APD, explained by activation of non-specific cation currents within the myocardium which shorten APD20 and lengthen APD80 see review by (Peyronnet et al., 2016). However, we saw no evidence of stretch-induced APD triangulation. An increase in dispersion of repolarisation can be pro-arrhythmic (Reiter et al., 1988) but we saw no evidence for this.

Stretch-induced ectopics were multi-focal in nature with no consistent pattern, pseudo-ECG recordings revealed single, double and short runs of ectopics (e.g., Figure 5B) suggestive of no single ectopic source or mechanism. We did not observe more serious arrhythmias, such as sustained tachycardia or fibrillation, even when an ectopic fell close to the peak of the t-wave of the preceding ECG (see Figure 5A). Such timing has been shown to be critical for the triggering of serious arrhythmias in *commotio cordis,* where mechanical stimulation occurs at the epicardial myocardium (Link and Estes, 2007; Quinn et al., 2017).

Optical mapping was used to investigate ectopic activation patterns. Currently, this technique requires the use of a contractile uncoupler, such as blebbistatin, to negate motion artefacts. This clearly affects the mechanical state of the myocardium. However, despite this limitation, the stretch-activated phenomenon under investigation was present in the presence of blebbistatin.

The profile of epicardial activation is dependent upon the source of endocardial stimulation (Martinez et al., 2018). Sinus stimulation involves excitation conducted through the PF network and conduction through the myocardium alone is incompatible with an activation pattern that replicates activation following sinus stimulation. Mechanically-induced ectopics with activation patterns closely resembling sinus-mediated activation most likely represent stimulation of the PF network close to the His bundle, conducting through the majority of the PF network. When the associated diastolic period is short, tissue refractoriness may slow conduction (e.g., Figure 5A excitation 3; Figure 5B excitation 6). The more distal to the His the site of PF stimulation becomes, the less co-ordinated early activation will be, as both forward and retrograde PF conduction take place (Haïssaguerre et al., 2016). However, we did observe ectopic activations that resembled focal endomyocardial stimulation (Figure 5A excitation 4).

In support of this interpretation of our observations, *in silico* modelling using a validated rabbit model with accurate PF and myocardial coupling and anatomy (Behradfar et al., 2014) predicts PF conduction will occur in response to endomyocardial foci *via* retrograde conduction in proximal PF-muscle junctions and that PF propagation will outpace that in the myocardium. It has recently been demonstrated that focal ectopic myocardial excitation can excite proximal PFs (Blackwell et al., 2022).

Therefore, we interpret our data as evidence for the involvement of the PF network in stretch-induced ectopics but not as evidence for the exclusion of a role for the endomyocardium. The precise role that PFs play, whether as ectopic source and/or substrate for excitation propagation, remains to be clarified in this model. A limitation of our study was that in order to derive the benefits of an intact heart model, it was not possible to image the endocardial surface during mechanical stimulation.

Recent observations in rat (Hurley et al., 2023) support the conclusion of this study. Lugol, decreased the number of stretch-activated arrhythmias in rats by 93% (compared with a 98% reduction in this study). However, ectopics were elicited by 100% of stretches in rats compared with 70% in rabbits and in the rat we saw periods of brief ventricular tachycardia that were not seen in the rabbit. The reasons for these species differences are currently unknown. The current study now demonstrates the importance of Purkinje fibres to acute dilation-induced arrhythmias in a species with a larger heart, slower heart rate and typical ventricular action potential profile (compared with rat) all important factors in arrhythmias. Furthermore we have supported our experimental observations with *in silico* predictions from a rabbit-specific model.

While the acute stretch model is not targeted to a specific pathology, and direct translation is not claimed, it can reveal mechanistic insights, such as a role for PFs, that may be relevant when considering mechanically-induced arrhythmias that occur in clinical settings where there is dilation, such as hypertrophy, heart failure and mitral regurgitation (Basso et al., 2019; Quinn and Kohl, 2021).

### 4.3 Conclusions

We conclude PFs do play a role in acute stretch/dilation-induced ectopics.

## Data Availability

The raw data supporting the conclusion of this article will be made available by the authors, without undue reservation.
