# Supplementary figures and images for: Attenuation of stretch-induced arrhythmias following chemical ablation of Purkinje fibres, in isolated rabbit hearts
Source: Front Physiol. 2023 Apr 6;14:1154157. doi: 10.3389/fphys.2023.1154157 (PMC10115947; doi:10.3389/fphys.2023.1154157)

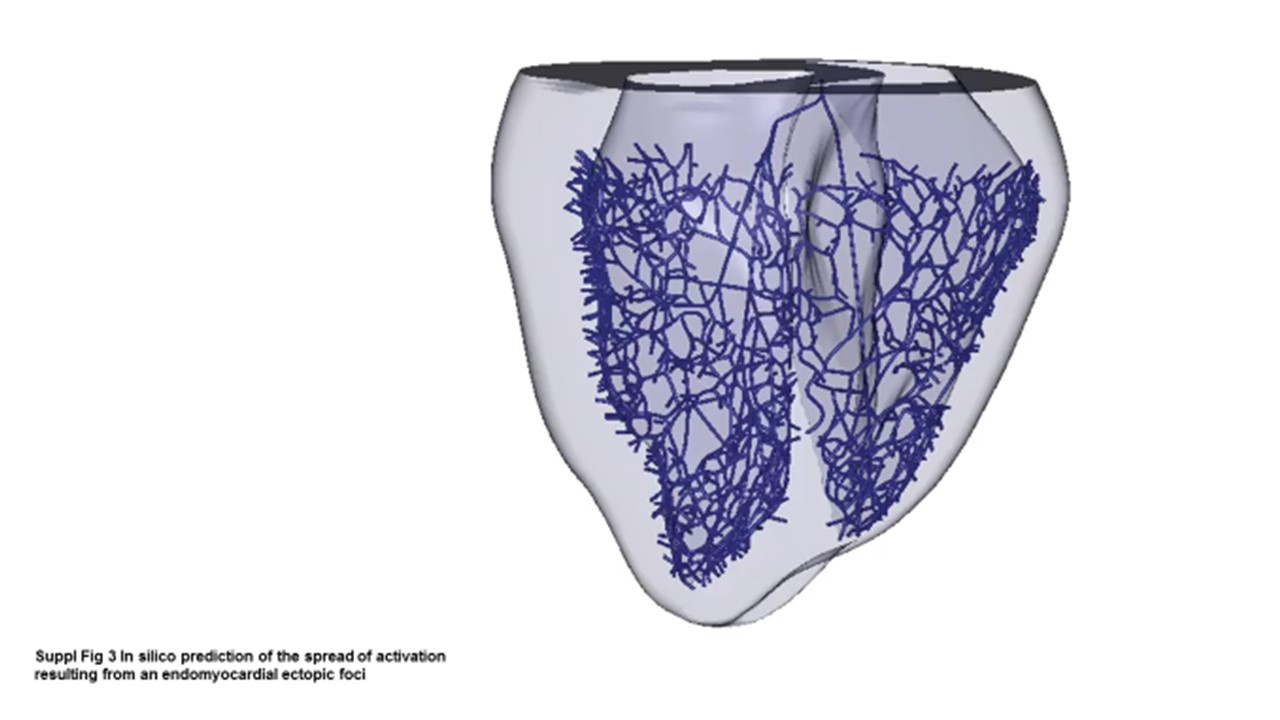

Supplement: Supplementary file 1 [file Image3.JPEG]

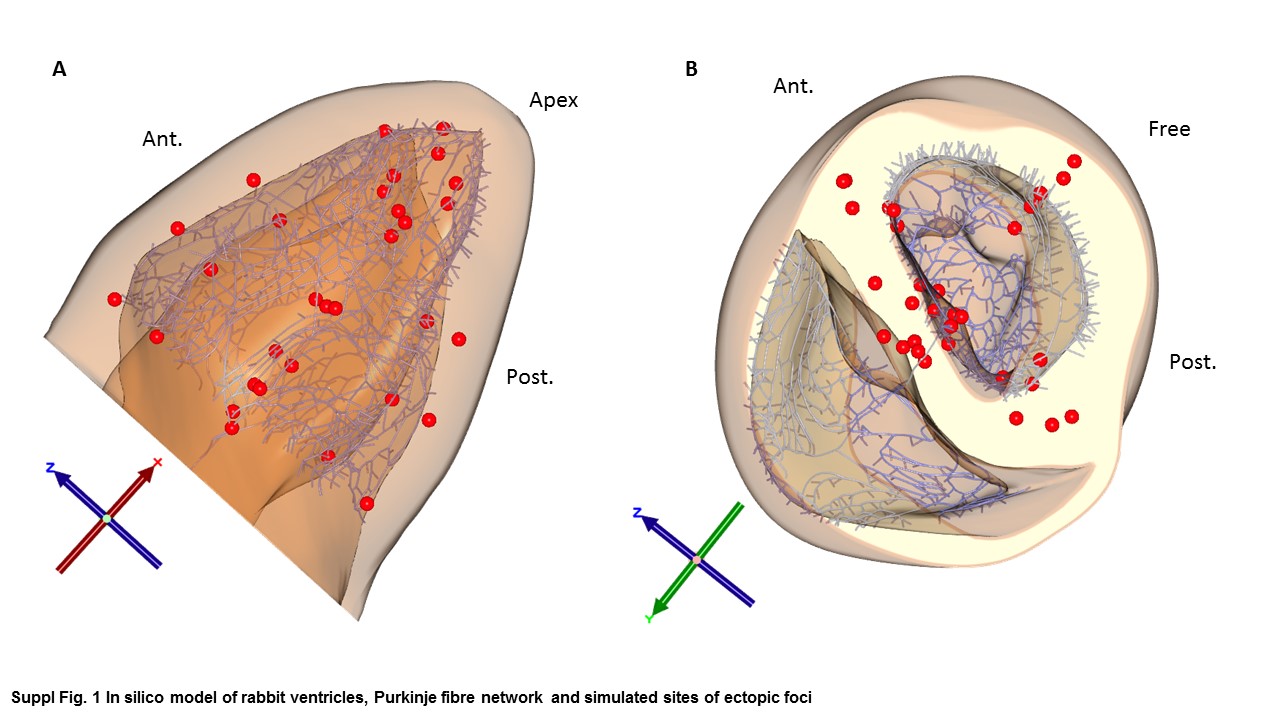

Supplement: Supplementary file 2 [file Image1.JPEG]

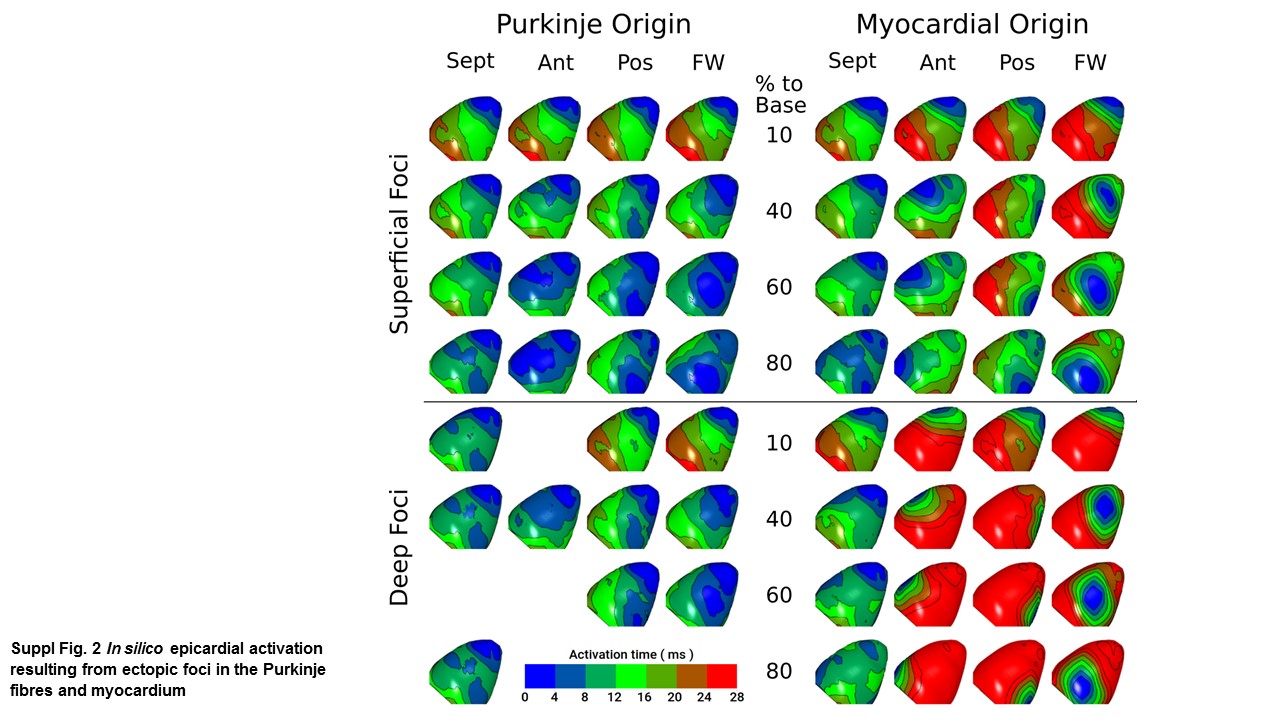

Supplement: Supplementary file 3 [file Image2.JPEG]
