# Supplementary material for: Attenuation of stretch-induced arrhythmias following chemical ablation of Purkinje fibres, in isolated rabbit hearts
Source: Front Physiol. 2023 Apr 6;14:1154157. doi: 10.3389/fphys.2023.1154157 (PMC10115947; doi:10.3389/fphys.2023.1154157)
Supplement: Supplementary file 5 [file DataSheet1.docx]

**Supplementary Methods and Results**

A finite element model of rabbit ventricles with a functional PF network (1-3) was used to simulate the propagation of ectopic activations. Ectopic locations from 32 sites in the LV were investigated in a 4*4*2 array. These sites were chosen by the researchers in order to give a wide coverage of foci sites in the LV. They were 10, 40, 60 and 80% of the distance from the apex to the base (4 levels), at septal, posterior, anterior and free wall locations (4 locations around the short axis) and on the endocardial surface (superficial foci) or 70% of the way to the epicardium (deep foci) (Suppl Fig. 1).

Each ectopic site was a 2 mm cube, and either only myocardium or only PF within each site was excited, for a total of 64 simulations. For each simulation, the first activation on the mapped surface was time 0 ms and all activation times are relative to that. Activation times were coloured with the colour map used in the optical mapping studies. Activation was identified by exceeding a transmembrane voltage threshold. The resultant epicardial activation maps are given in Suppl Fig. 2. For points near the base it was possible that no PFs were found within the cube, so there is no activation map. The closest match to sinus activation was for deep, PF septal stimulation, where the bundle branches are directly stimulated.

The results show that for myocardial foci, there is a centrifugal activation directly above the focus, and later breakthroughs distant from the site, resulting from activity which retrogradely entered and then exited the Purkinje system ahead of the advancing wavefront. For Purkinje ectopy, centrifugal activity was not seen and activation was strongly determined by the Purkinje junctions. Suppl Fig 3 is a video of whole ventricular activation resulting from an ectopic foci in the endomyocardium. Key points indicated in the figure legend demonstrate propagation through the Purkinje system outpacing that from the myocardial focus.

**Figure Legends**

**Supplemental Figure 1: In silico model of rabbit ventricles, Purkinje fibre network and simulated sites of ectopic foci**

(A) Longitudinal axis image of the LV with the septal wall removed to reveal the His-Purkinje system and sites of 2 mm cubic ectopic foci (red discs). Foci were from the endocardial surface (superficial foci) or from the myocardial region, 70 % the distance from the endocardial surface to the epicardial surface (deep foci). There were 4 stimulation sites around the LV: septal wall, anterior LV (Ant), posterior LV (Pos) and LV free wall (Free) and 4 longitudinal sites 10, 40, 60, 80 % the distance from apex to base. (B) Short axis image of the LV and RV with LV foci and Purkinje fibres indicated.

**Supplemental Figure 2: *In silico* epicardial activation resulting from ectopic foci in the Purkinje fibres and myocardium**

*In silico* epicardial activation maps in response to ectopic foci from 32 sites in the LV. Maps have 4 ms colour coded contours. Foci had either a Purkinje origin or a myocardial origin, and were from the endocardial surface (superficial foci) or from the myocardial region, 70 % of the distance from the endocardial surface (deep foci). There were 4 stimulation sites around the LV (columns), septal (Sept), anterior (Ant), posterior (Post) and free wall (FW) and 4 longitudinal sites (rows) 10, 40, 60, 80 % the distance from apex to base. Some deep foci sites lacked Purkinje fibres and do not give rise to activation. The image aside the calibration bar is the closest representation of sinus stimulation (a deep foci with Purkinje origin on the septal wall, 40% from the base, representing direct stimulation of the His-bundle branches).

The simulation predicts that the fastest spread of activation occurs in response to Purkinje foci, followed by activation in response to endomyocardial superficial foci. Deep myocardial foci predict activation patterns slower than those seen in optical experiments (compare Suppl. Fig 2 with Figs, 5a & 5b). The simulation shows that endomyocardial foci elicit depolarisations that travel to and propagate within the PF network whilst deep septal stimulation can result in fast activation as a result of bundle branch stimulation

**Supplemental Figure 3: In silico prediction of the spread of activation resulting from an endomyocardial ectopic foci**

Video of ventricular activation in an *in silico* model of rabbit LV and RV with anatomically accurate His-Purkinje network. The file has 61 frames (1 ms per frame, 0 to 60 ms). A 2 mm cubic ectopic foci in the LV endomyocardium leads to visible depolarisation after 3 ms. By 12 ms into the simulation, depolarisation is visible in the Purkinje network in advance of myocardial depolarisation at the site of the ectopic foci and by 18 ms this has given rise to myocardial depolarisation remote from the ectopic site. The model predicts that endomyocardial foci will propagate through the Purkinje fibre network.

**References**

1. Behradfar E, Nygren A, Vigmond EJ. The role of Purkinje-myocardial coupling during ventricular arrhythmia: a modeling study. PloS one. 2014;9(2):e88000.

2. Campos FO, Shiferaw Y, Prassl AJ, Boyle PM, Vigmond EJ, Plank G. Stochastic spontaneous calcium release events trigger premature ventricular complexes by overcoming electrotonic load. Cardiovascular research. 2015;107(1):175-83.

3. Campos FO, Shiferaw Y, Vigmond EJ, Plank G. Stochastic spontaneous calcium release events and sodium channelopathies promote ventricular arrhythmias. Chaos (Woodbury, NY). 2017;27(9):093910.
